# The Missing Link: Improving Cancer Outcomes Through a Structured Nutritional Quality Improvement Program

**DOI:** 10.7759/cureus.108299

**Published:** 2026-05-05

**Authors:** Shamael Albagar, Dalal S Salem, Ehab Hanafy, Sarah Al-Barraq, Sara Al Mousa, Faiez AlShafa

**Affiliations:** 1 Clinical Nutrition, Armed Forces Hospital Southern Region, Khamis Mushayt, SAU; 2 Oncology, Armed Forces Hospital Southern Region, Khamis Mushayt, SAU; 3 Pediatrics, Armed Forces Hospital Southern Region, Khamis Mushayt, SAU; 4 Quality Improvement, Armed Forces Hospital Southern Region, Khamis Mushayt, SAU; 5 Radiation Oncology, Armed Forces Hospital Southern Region, Khamis Mushayt, SAU

**Keywords:** cancer, malnutrition, nutritional support, oncology, oral nutritional supplements, pdsa, quality improvement, weight stability

## Abstract

Background

Malnutrition and weight instability are common but often under-recognized in patients receiving active cancer treatment. Treatment toxicities, altered metabolism, and psychological distress may reduce intake, promote muscle wasting, and cause unintentional weight loss, thereby worsening treatment tolerance, hospitalization risk, survival, and patient-reported outcomes. At the Armed Forces Hospital Southern Region (AFHSR) Oncology Center, improving nutritional care was identified as a patient safety and quality priority.

Local problem

A pre-intervention review of 50 patients receiving active oncologic therapy showed that nine (18.0%) experienced weight loss greater than 3%, three (6.0%) experienced weight gain greater than 3%, and 38 (76.0%) maintained stable body weight. These findings highlighted a significant care gap in systematic nutritional screening, timely referral, and proactive management of nutrition-related symptoms.

Methods

A structured quality improvement approach was used. Root cause analysis identified key contributors to unintentional weight loss, and Pareto analysis prioritized the most important and modifiable causes. A solution selection matrix was then applied to identify interventions that were feasible, cost-effective, and likely to yield meaningful clinical benefit. The selected strategies were implemented and refined through iterative FOCUS-PDSA cycles. Data analysis was conducted using the IBM SPSS Statistics for Windows, Version 27 (Released 2019; IBM Corp., Armonk, New York, United States).

Interventions

Three consecutive PDSA cycles were implemented in the Oncology and Radiation Departments at AFHSR. The first cycle focused on early nutritional screening and structured education for all eligible patients, with follow-up every two to three weeks. The second cycle targeted patients who did not maintain stable body weight despite education alone; these patients received individualized dietary counselling, oral nutritional supplementation, and practical guidance regarding meal size, content, and timing. The third cycle addressed refractory cases with persistent weight loss risk despite prior interventions and incorporated pharmacological management of chemotherapy-induced nausea and vomiting as well as psychological support to improve adherence and nutritional intake.

Results

A total of 141 patients were enrolled, of whom 117 (83%) received chemotherapy. Although all 141 patients received nutritional education, 82 (58%) required oral nutritional supplements, 106 (75%) maintained stable body weight, and 35 (25%) required pharmacologic intervention. Control charts showed improvement in weight stability from about 80% initially to a mean of 93.6%, within control limits and without rule violations. Moving range charts showed reduced variability over time. Process capability was high (Cp=2.809, Cpk=2.541). Analyses of >3% weight loss and gain also showed capable, well-centered processes. Patient satisfaction with nutritional education, oral supplementation, and the overall nutrition service was high.

Conclusions

A structured multidisciplinary nutritional support program significantly improved and sustained weight stability among patients receiving active cancer treatment. The intervention achieved statistical control, high process capability, and consistent adherence, supporting its value as a cost-effective and sustainable quality improvement strategy in oncology practice.

## Introduction

Problem description

Malnutrition and weight instability are common yet under-recognized problems among patients receiving active cancer treatment. During chemotherapy, radiotherapy, or concurrent chemoradiation (CCRT), treatment-related toxicities such as nausea, vomiting, diarrhea, mucositis, and dysgeusia frequently reduce oral intake and contribute to clinically significant weight loss [1,2]. In addition, malignancy and its treatment may induce a hypermetabolic, catabolic state that accelerates protein breakdown and promotes progressive muscle wasting and cachexia [3]. Psychological distress, including anxiety, depression, and fear, may further suppress appetite and reduce adherence to dietary recommendations, thereby worsening nutritional decline [4].

These changes are clinically important because malnutrition and weight instability are associated with poorer survival, lower patient-reported quality of life, higher treatment-related toxicity, and increased unplanned hospitalizations [5]. Nutritional deterioration during active cancer therapy may also contribute to reduced functional status, treatment interruptions, delayed completion of therapy, and increased healthcare costs [5]. At the Armed Forces Hospital Southern Region (AFHSR) Oncology Center, improving nutritional care was therefore identified as an important patient-safety and quality-of-care priority.

Available knowledge

Nutrition is increasingly recognized as a core component of oncology care rather than an optional supportive measure. International guidance recommends routine malnutrition screening, early dietetic assessment, and proactive management of symptoms that interfere with intake, as weight loss, sarcopenia, and inflammation are consistently associated with poorer outcomes [6,7]. Previous studies have shown that nutritional deterioration during chemotherapy or radiotherapy is associated with inferior treatment tolerance, lower treatment completion, and worse patient-centered outcomes [8].

Body composition studies have further shown that sarcopenia may increase the risk of toxicity independently of body mass index, highlighting that nutritional care should focus not only on body weight but also on the preservation of lean mass and functional reserve [9]. Interventional evidence suggests that individualized dietary counselling and oral nutritional supplementation (ONS) can improve weight maintenance, treatment tolerance, appetite, and quality of life compared with usual care [10]. In selected oncology populations, early structured nutritional pathways have also been associated with reduced treatment-related weight loss, fewer infections, and shorter hospitalization [11]. More recently, integrative nutritional models embedded within multidisciplinary oncology workflows have been proposed as practical strategies to improve treatment continuity and patient outcomes through routine screening, timely referral, standardized nutritional intervention, and symptom-directed supportive care [12,13].

Rationale

At AFHSR, baseline review of 50 consecutively managed oncology patients demonstrated that although 76% maintained stable body weight during active treatment, 18% experienced weight loss greater than 3%, while 6% experienced weight gain greater than 3%. These findings identified a significant local gap in systematic nutritional screening, timely referral, and proactive management of nutrition-related symptoms. Because nutritional risk may emerge early and progress silently during active therapy, a structured and proactive approach was considered necessary.

The rationale for this project was that unintended weight loss in oncology patients is driven by a combination of modifiable biological, symptomatic, and psychosocial factors. Treatment-related toxicities reduce intake, cancer-related hypermetabolism and inflammation accelerate catabolism, and psychological distress interferes with appetite and adherence. A pathway that addresses these factors together through routine screening, individualized education, early dietitian involvement, ONS, and coordinated supportive management was therefore expected to improve weight stability and strengthen treatment tolerance. In this context, nutrition was approached not as an isolated supportive measure, but as a practical quality and safety strategy embedded within routine oncology care.

Specific aims

This project aimed to reduce the percentage of patients experiencing weight loss of ≥3% during active oncologic management, including chemotherapy, CCRT, or radiation therapy, from a baseline rate of 18% to 8% by the third quarter of 2025. This timeline was selected to allow sequential implementation, monitoring, and refinement of the structured nutritional pathway across three Plan, Do, Study, Act (PDSA) cycles, including nutritional education, ONS, and pharmacologic escalation for selected refractory cases.

The primary objective was to reduce clinically significant weight loss during active treatment. Secondary objectives were to improve the proportion of patients maintaining stable body weight within ±3% of baseline, increase standardized nutritional counselling and timely escalation to ONS, monitor unintended weight gain as a balancing measure, and evaluate patient satisfaction with the nutrition service.

## Materials and methods

Context

This quality improvement project was conducted at the Oncology Center of the AFHSR, Saudi Arabia, including the Chemotherapy and Radiation Therapy departments. Patients with histopathologically confirmed malignancies were consecutively enrolled starting from their first treatment cycle and were followed prospectively throughout active treatment. Assessments were performed during every treatment cycle, typically at approximately three-week intervals, through direct clinical evaluation, nutritional assessment, and multidisciplinary follow-up in both outpatient and virtual clinic settings.

This was a prospective quality improvement project, not a randomized controlled trial. Patients were enrolled consecutively during routine oncology care, and the intervention was implemented as a structured service-improvement pathway using sequential Find, Organize, Clarify, Understand, Select-Plan, Do, Study, Act (FOCUS-PDSA) cycles. Nutritional risk was assessed pragmatically at each treatment visit using weight trajectory, oral intake history, treatment-related symptoms affecting intake, dietitian assessment, and the presence of clinically significant weight loss, defined as a change of ≥3% from baseline body weight. Body weight was recorded at baseline and at subsequent treatment visits using clinic-based weighing scales, with the percentage weight change calculated relative to baseline. Patients with stable weight within ±3% of baseline continued standardized nutritional education and follow-up, whereas patients with ≥3% weight loss, reduced intake, poor appetite, or significant nutrition-impact symptoms were escalated to individualized dietitian counselling and ONS.

The study population comprised adult patients with malignant disease confirmed by histopathological examination between April 2025 and September 2025. All participants underwent routine weight assessment, nutritional follow-up, and evaluation of adherence to nutritional counselling and supplement use. Compliance with dietary education, utilization of prescribed nutritional support, and attendance at virtual nutritional consultations were also monitored to assess continuity of nutritional care throughout the treatment period. Eligible patients were aged 18 years or older, had histopathological confirmation of malignancy, were undergoing active oncologic management including chemotherapy and/or radiotherapy, and were expected to complete at least two consecutive treatment cycles within the study timeframe. Exclusion criteria were receipt of palliative or best supportive care only without active oncologic therapy, concurrent enrolment in another interventional clinical trial that could influence nutritional or weight outcomes, severe cognitive impairment or communication barriers precluding reliable data collection, and pregnancy or lactation.

The project was delivered by a cross-functional multidisciplinary team comprising nutrition and dietetics, oncology, radiotherapy, nursing, and quality representatives, with defined operational and oversight.

**Figure 1:**
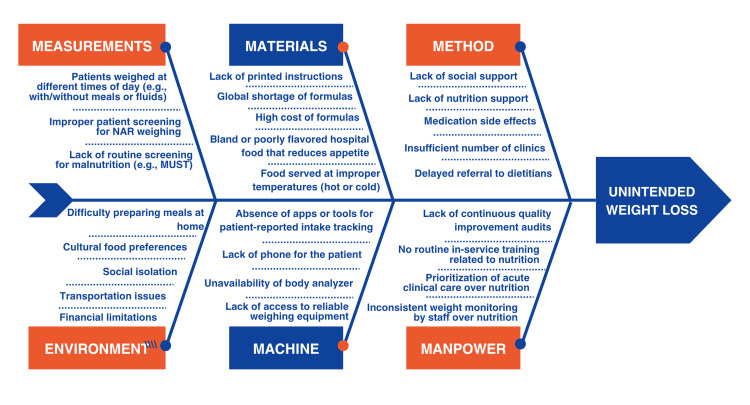
Fishbone analysis of contributors to unintended weight loss in patients receiving active cancer treatment. Cause-and-effect diagram showing the main contributing factors to unintended weight loss across measurement, materials, methods, machine, manpower, and environmental domains. NAR: nutritional at-risk; MUST: Malnutrition Universal Screening Tool Image credit: The image was created by the authors using Microsoft PowerPoint (Microsoft Corp., Redmond, WA, USA).

To understand the local care gap and intervention context, a root cause analysis was performed. Fishbone analysis identified multifactorial contributors to unintended weight loss, including inconsistent weighing practices, absence of standardized malnutrition screening, limited nutritional formulas, delayed dietitian referral, inadequate nutrition support, treatment-related side effects, social and financial barriers, limited staff training, shortage of dietitians, poor interdisciplinary communication, unreliable weighing equipment, and lack of digital tracking tools. These findings are summarized in Figure 1.

**Figure 2:**
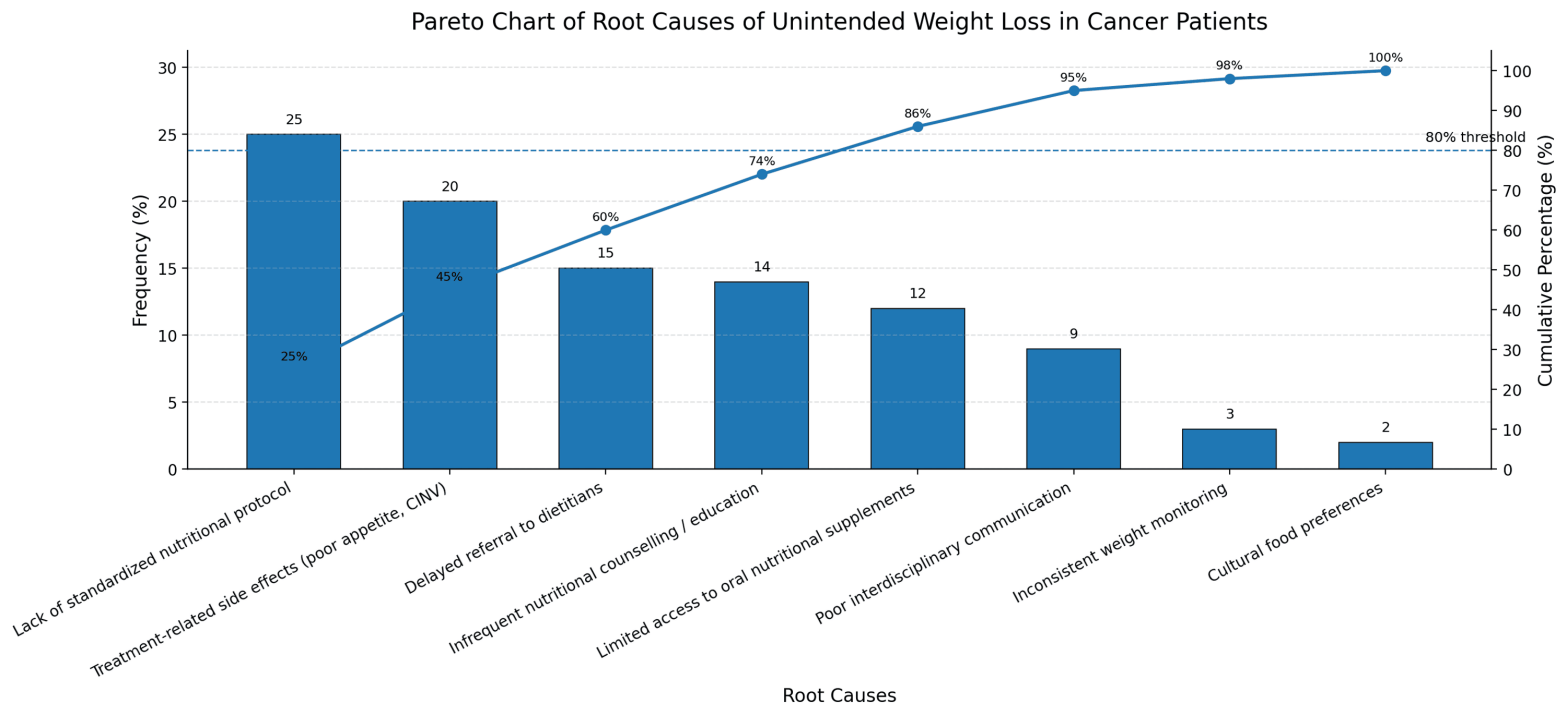
Pareto chart of root causes contributing to unintended weight loss in patients with cancer. Pareto analysis based on the baseline review of 50 patients showing the relative frequency and cumulative contribution of identified root causes. The 80% threshold is reached by the first five factors, led by lack of a standardized nutritional protocol, treatment-related side effects, delayed referral to dietitians, infrequent nutritional counselling, and limited access to oral nutritional supplements. CINV: chemotherapy-induced nausea and vomiting Image credit: The image was created by the authors using Microsoft PowerPoint (Microsoft Corp., Redmond, WA, USA).

Pareto analysis was subsequently used to prioritize the most influential causes. The leading contributors were a lack of standardized nutritional screening, inadequate dietary counselling and education, delayed referral to dietitians, treatment-related side effects including poor appetite and chemotherapy-induced nausea and vomiting, and limited access to ONS. Together, these five factors accounted for approximately 86% of the problem and were therefore selected as priority targets for intervention. The Pareto findings are shown in Figure 2.

**Figure 3:**
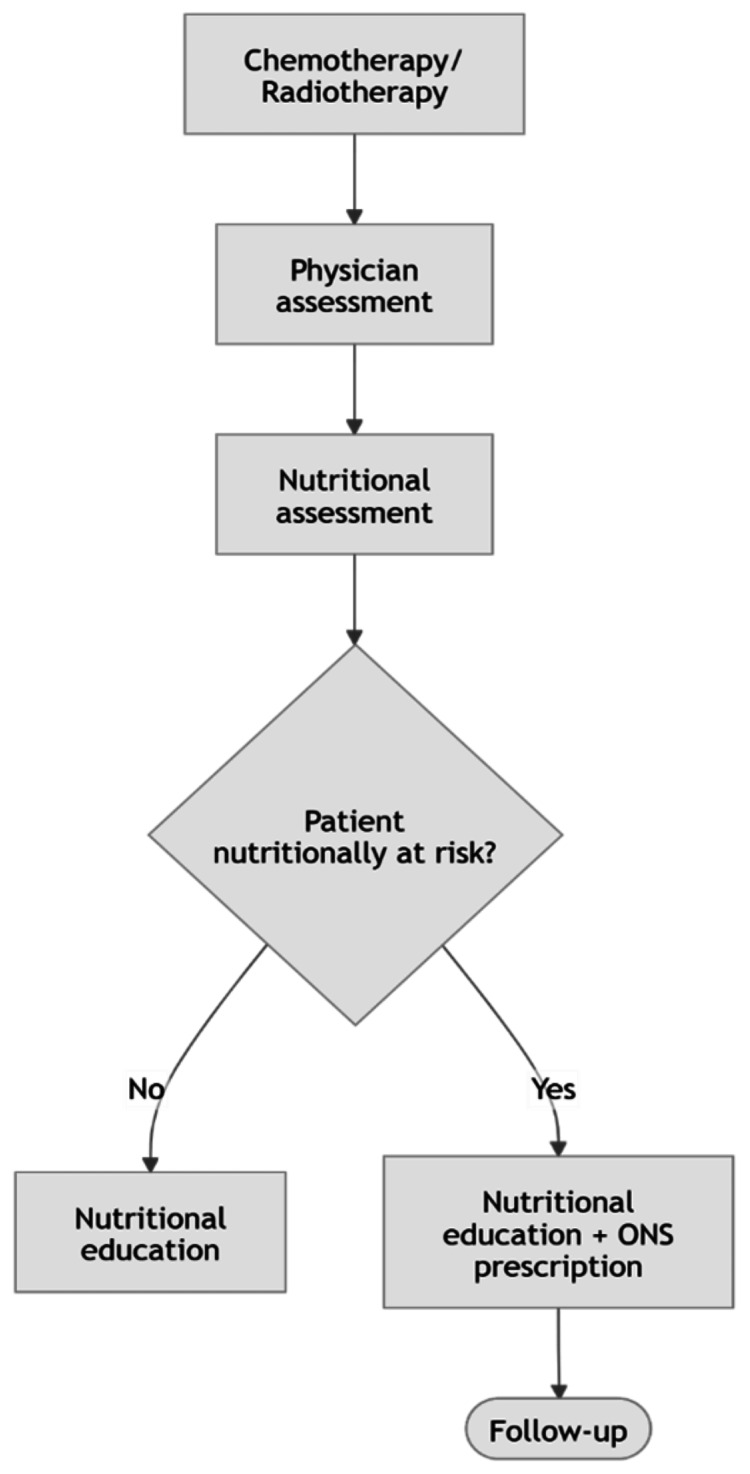
Proposed nutrition screening and intervention pathway for patients receiving cancer treatment. Clinical workflow showing physician and nutritional assessment, risk stratification, provision of nutritional education for all patients, oral nutritional supplements (ONS) for those at nutritional risk, and follow-up after intervention. Image credit: The image was created by the authors using Mermaid (Mermaid JS, Copenhagen, Denmark).

Baseline workflow review demonstrated that nutritional management before the project was reactive and referral-based rather than standardized and proactive. The pre-intervention process began with physician assessment, followed by selective nutritional assessment and counselling only when a specific issue was recognized, with ONS prescribed according to individual clinical judgment and follow-up arranged thereafter. This baseline process is illustrated in Figure 3.

Intervention(s)

A structured solution selection matrix was used to prioritize candidate interventions according to their potential to meet project goals, positive patient impact, implementation cost, stakeholder buy-in, and time feasibility using weighted scoring. The highest-ranked interventions were the establishment of a specialized oncology-nutrition clinic and the development of a standardized nutrition support protocol for oncology patients, followed by allocation of a dedicated dietitian and implementation of remote nutritional support. The solution prioritization matrix is presented in Table 1.

**Table 1:** Prioritization matrix of proposed interventions to address unintended weight loss in patients with cancer. Weighted scoring was based on projected goal achievement, positive patient impact, implementation cost, stakeholder buy-in, and time to implementation. Higher scores indicate greater overall priority for implementation. * Implementation cost: 1 = very high cost; 5 = very low cost. ^† ^Time to implement: 1 = long implementation time; 5 = rapid implementation.

Potential solution	Potential to meet project goal (weight 10)	Positive patient impact (weight 9)	Implementation cost* (weight 8)	Stakeholders buy-in (weight 7)	Time to implement^†^ (weight 5)	Total weighted score	Selected to implement
Provide a wider variety of nutritional formulas for patients to choose from	5	5	2	4	3	154	Yes
Establish a specialized oncology nutrition clinic	5	5	5	5	5	195	Yes
Develop a standardized nutrition support protocol for oncology patients	5	5	5	5	5	195	Yes
Ensure the availability of appropriate weighing scales for ambulatory and non-ambulatory patients	4	3	2	5	2	128	Yes
Allocate a specialized dietitian within the oncology center	5	5	3	4	5	172	Yes
Implement remote nutritional support through virtual consultations and home-delivered supplements	5	5	2	4	5	164	Yes

**Figure 4:**
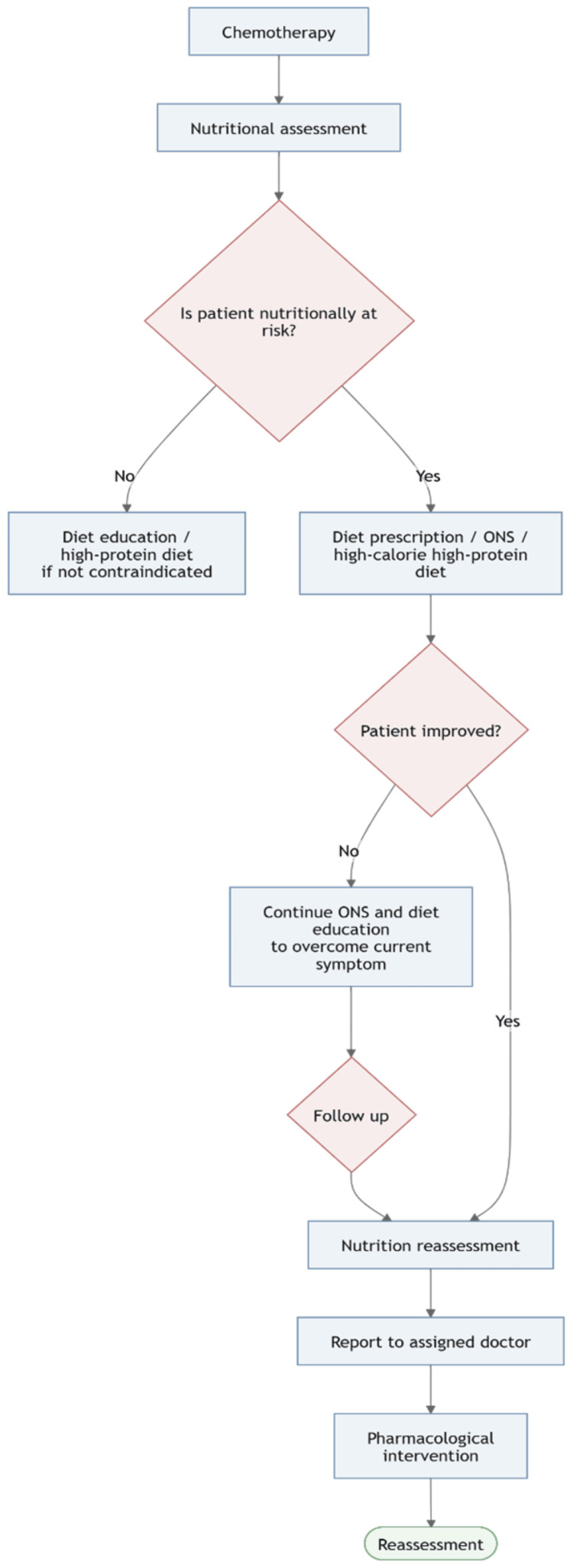
Post-implementation nutrition care pathway for patients receiving chemotherapy. Updated workflow showing nutritional risk screening, diet education for patients not at risk, prescription of ONS, and a high-calorie, high-protein diet for at-risk patients, follow-up with reassessment, reporting to the assigned physician, and pharmacological intervention when needed. ONS: oral nutritional supplements Image credit: The image was created by the authors using Mermaid (Mermaid JS, Copenhagen, Denmark).

Based on this prioritization, a structured integrative nutrition pathway was implemented for patients receiving active oncologic treatment. The intervention flow began with nutritional assessment after treatment initiation. Patients not considered nutritionally at risk received diet education, including high-protein dietary advice when not contraindicated. Patients identified as nutritionally at risk received diet prescription, ONS, and continued follow-up. Those who did not improve were reassessed, reported to the assigned physician, and escalated to pharmacological intervention, followed by further reassessment. This intervention flow is shown in Figure 4.

ONS was prescribed as part of individualized dietitian-led care rather than as a fixed research product. The choice of supplement formulation, frequency, and duration was determined according to nutritional risk, oral intake, tolerance, taste preference, comorbidities, and product availability. Patients were counselled regarding supplement timing, meal spacing, high-calorie and high-protein food choices, and strategies to manage nutrition-impact symptoms. Adherence and tolerance were reviewed during follow-up visits, and the plan was modified when patients reported poor palatability, gastrointestinal intolerance, or inadequate intake.

**Figure 5:**
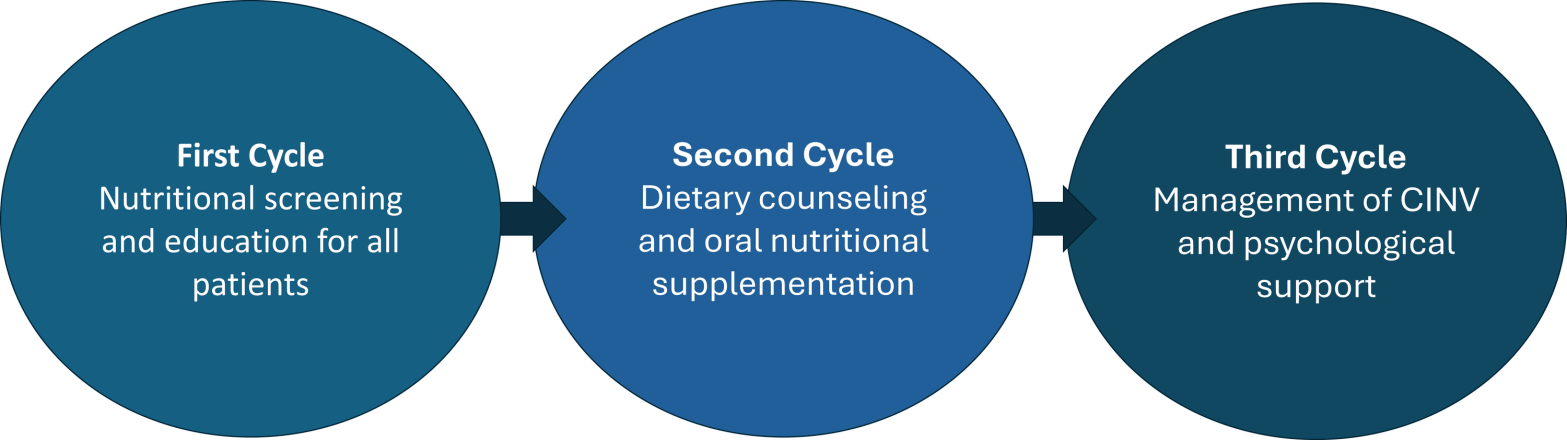
Stepwise nutritional intervention across chemotherapy cycles. Sequential model showing nutritional screening and education during the first cycle, dietary counselling with oral nutritional supplementation during the second cycle, and management of chemotherapy-induced nausea and vomiting with psychological support during the third cycle. CINV: chemotherapy-induced nausea and vomiting Image credit: The image was created by the authors using Microsoft PowerPoint (Microsoft Corp., Redmond, WA, USA).

The project introduced three main interventions, implemented and refined through sequential PDSA cycles, as summarized in Figure 5. The first intervention focused on nutritional education, using standardized patient-friendly educational materials and reinforcement of dietary counselling at each clinic visit. The second targeted higher-risk patients through ONS to better meet caloric and protein requirements. The third introduced pharmacological support, led by oncology physicians, using agents such as olanzapine to improve appetite, control delayed chemotherapy-induced nausea and vomiting, and support mood, thereby contributing to weight stability.

The intervention was implemented across three sequential PDSA cycles within the study period from April 2025 to September 2025. PDSA cycle 1 (nutritional education) was conducted from 1 April 2025 to 31 May 2025; PDSA cycle 2 (ONS) from 1 June 2025 to 31 July 2025; and PDSA cycle 3 (pharmacological intervention) from 1 August 2025 to 30 September 2025.

PDSA cycle 1: nutritional education

The first cycle aimed to introduce a standardized nutritional education program for cancer patients receiving active treatment, with emphasis on practical strategies to maintain weight stability. During implementation, patients received educational leaflets, posters, and counselling at baseline, reinforced by staff at every clinic visit, and oncology staff were trained to deliver consistent messages. The cycle was evaluated through patient feedback regarding understanding of dietary advice, monitoring of weight change patterns, and review of staff compliance with educational reinforcement. Based on these observations, the educational component was integrated into routine care, and the materials were modified to better accommodate cultural and literacy needs.

PDSA cycle 2: ONS

The second cycle aimed to enhance nutritional support through the provision of ONS to patients identified as being at nutritional risk, particularly those with weight loss greater than 3%. Dietitians screened patients during clinic visits, prescribed supplements to at-risk patients, and educated them on correct usage. Adherence was monitored regularly. This cycle was studied by measuring the proportion of patients receiving ONS, their adherence, and the effect on weight stability, in addition to gathering patient feedback regarding palatability and tolerance. Following favourable results, ONS was incorporated into routine clinical protocols, with efforts to improve supply chain processes and diversify supplement options.

PDSA cycle 3: pharmacological intervention

The third cycle focused on patients with reduced appetite and persistent risk of weight loss despite prior interventions. The intervention centered on pharmacological strategies prescribed by oncology physicians, particularly olanzapine, selected for its role in delayed control of chemotherapy-induced nausea and vomiting, appetite stimulation, and mood support. Eligible patients, such as those with poor appetite, nausea, or mood-related barriers contributing to weight loss, were prescribed treatment according to clinical guidance and were monitored during follow-up for tolerance, adverse effects, and adherence. This cycle was evaluated by tracking appetite, weight change, and quality-of-life-related indicators, and by comparing outcomes in patients managed with versus without pharmacological support. When results suggested benefit, olanzapine use was standardized within supportive oncology protocols, with ongoing review of indications and dosing for safety and appropriateness.

Study of the intervention(s)

The intervention was studied as an iterative, real-world quality improvement process embedded in routine oncology care. Each PDSA cycle was evaluated in sequence to determine whether escalation from education alone to ONS and then to pharmacological support for selected patients resulted in measurable improvement in nutritional stability and reduction in clinically significant weight change. The sequential PDSA structure allowed refinement of the pathway over time and supported integration of successful changes into the standard workflow.

The measures used to study intervention performance are summarized in Table 2. These included indicators reflecting weight loss, weight stability, nutritional counselling, ONS, adherence to the nutritional plan, and balancing surveillance for unintended weight gain. The measure framework was used to evaluate whether the interventions improved outcomes while maintaining safety and consistency of care. Patient-reported experience and satisfaction with nutritional education, ONS, and the overall clinical nutrition service were also assessed using structured patient feedback tools.

**Table 2:** Project outcome, process, and balancing measures with operational definitions and performance benchmarks. Outcome measures evaluated weight loss and weight stability; process measures assessed delivery of nutritional interventions and patient adherence; the balancing measure monitored unintended weight gain.

Measure	Measure type	Numerator	Denominator	Benchmark
Percentage of patients experiencing ≥3% weight loss during chemotherapy/radiation therapy	Outcome	Number of patients who lost ≥3% of baseline body weight	Total number of patients	≤10%
Percentage of patients maintaining stable body weight during chemotherapy/radiation therapy	Outcome	Number of patients with body weight within ±3% of baseline	Total number of patients	≥80%
Percentage of patients who received documented nutritional counselling during active treatment	Process	Number of patients who received documented nutritional counselling during active treatment	Total number of patients	≥90%
Percentage of nutritionally at-risk patients receiving oral nutritional supplements (ONS)	Process	Number of nutritionally at-risk patients receiving ONS as part of nutritional management	Total number of patients identified as nutritionally at risk	≥80%
Percentage of patients compliant with their nutritional plan	Process	Number of patients adherent to the prescribed dietary and ONS plan	Total number of patients provided with a nutritional plan	≥75%
Percentage of patients experiencing >3% unintentional weight gain	Balancing	Number of patients experiencing >3% unintentional weight gain	Total number of patients	≤10%

Measures

Quality improvement measures were categorized as process, outcome, and balancing measures in the source document. These included the percentage of patients experiencing at least 3% weight loss during chemotherapy and/or radiation therapy, the percentage maintaining stable body weight within ±3% of baseline, the percentage receiving documented nutritional counselling, the percentage of nutritionally at-risk patients receiving ONS, the percentage compliant with their nutritional plan, and the percentage experiencing unintentional weight gain greater than 3%. Predefined benchmark targets were specified for each measure and were aligned in the source document with international standards, including the European Society for Clinical Nutrition and Metabolism (ESPEN) and the National Comprehensive Cancer Network (NCCN) guidance [14,15]. These measures and their numerators, denominators, and targets are detailed in Table 2.

Analysis

Statistical analysis was performed using IBM SPSS Statistics for Windows, Version 27 (Released 2019; IBM Corp., Armonk, New York, United States), including the Quality Control Analysis function. Baseline clinical characteristics were summarized using frequencies, means, and percentages. Relative percentage weight change was calculated using the formula: (visit weight-baseline weight)/baseline weight × 100. Weight stability was defined as body weight remaining within ±3% of baseline, clinically significant weight loss as a decrease of ≥3%, and unintended weight gain as an increase of >3%. Missing data were handled using the Last Observation Carried Forward method to preserve longitudinal follow-up records.

To evaluate improvement over time and process stability, control charts were developed across sequential patient visits. Individual (I) charts and moving range (MR) charts were constructed to assess the percentage of patients experiencing clinically significant weight changes, defined as greater than 3% gain or loss of body weight. Control limits were determined according to the Six Sigma principle (±3 standard deviations), representing expected common-cause variation. Points outside these limits, or patterns violating standard control chart rules such as runs or trends, were interpreted as evidence of special-cause variation requiring further assessment. Process capability indices were also calculated to quantify process consistency and centering relative to predefined specification limits, including Cp, which reflects overall process capability; Cpk, which reflects process capability adjusted for centering; CpL, which reflects capability relative to the lower specification limit; and CpU, which reflects capability relative to the upper specification limit. This analytical approach was used to assess both improvement and sustainability of the nutritional intervention process over time.

Ethical considerations

This project was conducted in accordance with the ethical principles of the Declaration of Helsinki and institutional policies governing quality improvement activities. As the initiative aimed to improve patient care through nutritional and supportive interventions already consistent with routine clinical practice, no experimental treatment was introduced. Participation in weight monitoring and nutritional assessment was voluntary, and patients were informed of the project objectives and of the intended use of collected data for service improvement.

Patient confidentiality was maintained throughout. Identifiers were removed before analysis, data were aggregated to protect privacy, and access to clinical information was restricted to authorized members of the quality improvement team. Because the project focused on the evaluation and improvement of routine care processes, it was considered minimal risk.

## Results

A total of 141 patients with histopathologically confirmed malignancies were enrolled during the project period. Regarding treatment modality, the majority received chemotherapy (117/141, 83.0%), while 18/141 (12.8%) received CCRT and 6/141 (4.3%) received radiotherapy alone (RTH) (Table 3).

**Table 3:** Treatment modalities among patients included in the project. Data are presented as numbers and percentages of the total cohort (n=141).

Treatment	Modality	Count (n=141)	Percentage (%)
Modality of treatment	Chemotherapy	117	83.0
	Concurrent chemoradiation	18	12.8
	Radiation therapy only	6	4.3

**Figure 6:**
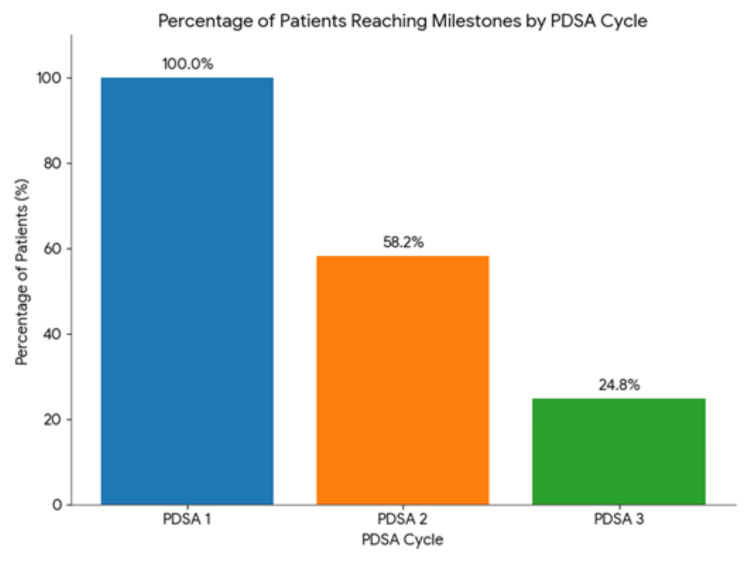
Percentage of patients progressing through each PDSA cycle of the nutritional intervention (n=141). Bar chart showing participation across sequential intervention phases: 141 (100.0%) patients received nutritional education in PDSA 1, 82 (58.2%) required oral nutritional supplementation in PDSA 2, and 35 (24.8%) progressed to PDSA 3, reflecting escalation of care according to nutritional risk and response to prior measures. PDSA: Plan, Do, Study, Act

Participation across the sequential intervention phases is summarized in Figure 6, which demonstrates the proportion of patients exposed to each PDSA cycle. All patients received the foundational educational intervention, while subsequent cycles reflected escalation of care according to nutritional risk and response to prior measures.

At the start of the intervention pathway, 100% of patients received nutritional education, establishing a standardized baseline for care delivery. Despite this universal educational input, 58.2% of patients required ONS, indicating that education alone was insufficient for a substantial proportion of patients undergoing active oncologic treatment. Overall, 75.2% of patients adhered to the nutritional plan and maintained stable body weight, whereas 24.8% did not maintain adequate adherence and a considerable proportion of these patients required escalation to pharmacologic support, including agents such as olanzapine, to address anorexia, treatment-related nausea and vomiting, and other barriers to intake. These findings are reflected in the project measure framework (Table 4) and the intervention participation chart (Figure 6).

**Table 4:** Delivery of nutritional interventions and compliance with the nutritional plan. Data are presented as numbers and percentages of the total cohort (n=141). Oral nutritional supplementation was prescribed according to nutritional risk assessment.

Intervention	Response	Count (n=141)	Percentage (%)
Nutritional education	Yes	141	100.0
	No	0	0.0
Oral nutritional supplement	Required	82	58.2
	Not required	59	41.8
Compliance with nutritional plan	Compliant	106	75.2
	Non-compliant	35	24.8

**Figure 7:**
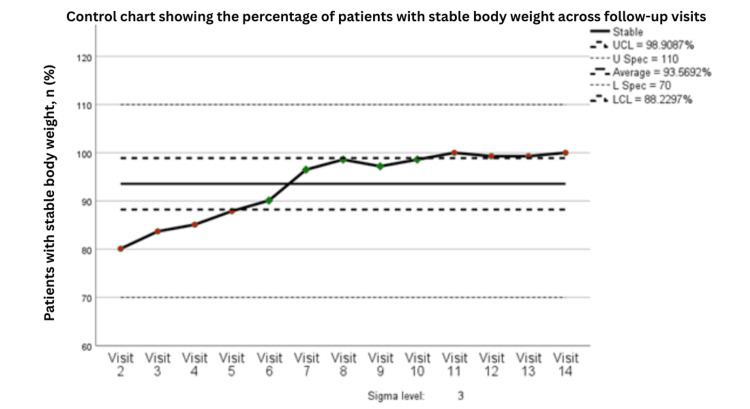
Control chart showing the percentage of patients with stable body weight across follow-up visits (n=141). Statistical process control chart showing the percentage of patients with stable body weight across follow-up visits, based on 141 patients at each time point. Visits 1-3 correspond to PDSA Cycle 1, Visits 4-6 correspond to PDSA Cycle 2, and Visits 7-9 correspond to PDSA Cycle 3. UCL indicates the upper control limit, LCL the lower control limit, U Spec the upper specification limit, and L Spec the lower specification limit. Green points indicate observations within expected process behavior, whereas red points indicate observations flagged by control chart rules as potential special-cause variation. PDSA: Plan, Do, Study, Act

The principal outcome of the project was progressive improvement in the proportion of patients maintaining stable body weight during active treatment. The control chart demonstrated that weight-stability rates were lower during earlier visits, at approximately 80% by Visit 2, but improved steadily over time. By Visit 7, the process exceeded the average performance level of 93.6% and remained close to the upper control limit of 98.9% during subsequent visits. All observed data points remained within control limits, with no rule violations, indicating a statistically stable and well-controlled process (Figure 7).

**Figure 8:**
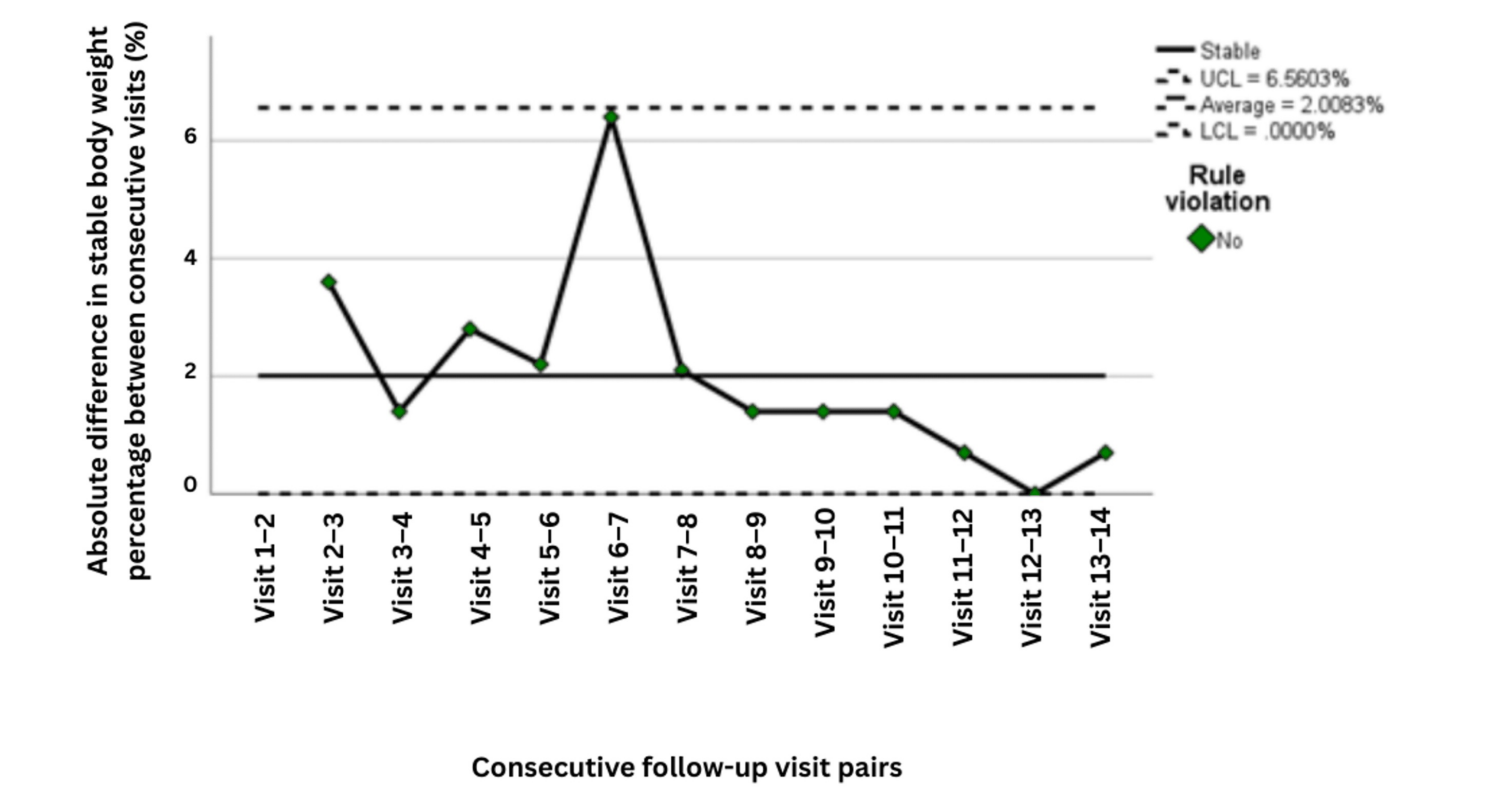
Moving range control chart for visit-to-visit variation in the percentage of patients with stable body weight across follow-up visits (n=141). Moving range control chart showing the absolute visit-to-visit variation in the percentage of patients with stable body weight, based on 141 patients at each time point. The moving range was calculated from pairs of consecutive visits (moving range of 2). Visits 1-5 correspond to PDSA Cycle 1, Visits 6-9 correspond to PDSA Cycle 2, and Visits 10-14 correspond to PDSA Cycle 3. Weight stability was treated as a categorical outcome at each visit, defined as body weight within ±3% of baseline, and the chart displays the variation in the percentage of patients meeting this criterion between consecutive visits. No control chart rule violations were detected, indicating no evidence of special-cause variation. The x-axis represents sequential pairs of consecutive follow-up visits. UCL indicates the upper control limit, and LCL indicates the lower control limit. PDSA: Plan, Do, Study, Act

The corresponding moving-range chart showed that variability in weight stability was greater during the earlier phase of implementation but declined progressively over later visits, indicating improved process consistency as the intervention became embedded within routine care (Figure 8). Process capability analysis further supported these findings, showing excellent performance with Cp = 2.809 and Cpk = 2.541, both well above the conventional benchmark of 1.33, confirming that the process was not only stable but also highly capable of maintaining performance within the predefined target range (Table 5).

**Table 5:** Process capability indices for weight stability during follow-up. ^a^ The normal distribution was assumed, with a lower specification limit (LSL) of 80 and an upper specification limit (USL) of 110. The estimated capability sigma was based on the mean of the sample moving ranges. Cp: process capability index; Cpl: lower process capability index; Cpu: upper process capability index; Cpk: minimum process capability index adjusted for process centering

Process statistics		
Capability indices	Cp^a^	2.809
	Cpl^a^	2.541
	Cpu^a^	3.077
	Cpk^a^	2.541

**Figure 9:**
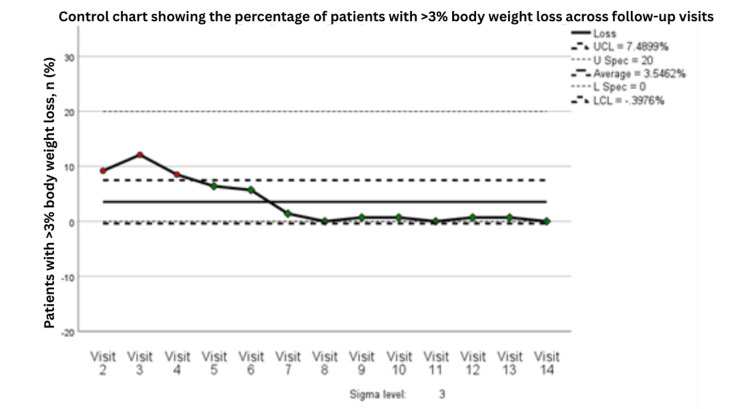
Control chart showing the percentage of patients with >3% body weight loss across follow-up visits (n=141). Statistical process control chart showing the percentage of patients with >3% body weight loss across follow-up visits, based on 141 patients at each time point. Visits 1-3 correspond to PDSA Cycle 1, Visits 4-6 correspond to PDSA Cycle 2, and Visits 7-9 correspond to PDSA Cycle 3. UCL indicates the upper control limit, LCL the lower control limit, U Spec the upper specification limit, and L Spec the lower specification limit. Green points indicate observations within expected process behavior, whereas red points indicate observations flagged by control chart rules as potential special-cause variation. PDSA: Plan, Do, Study, Act

**Figure 10:**
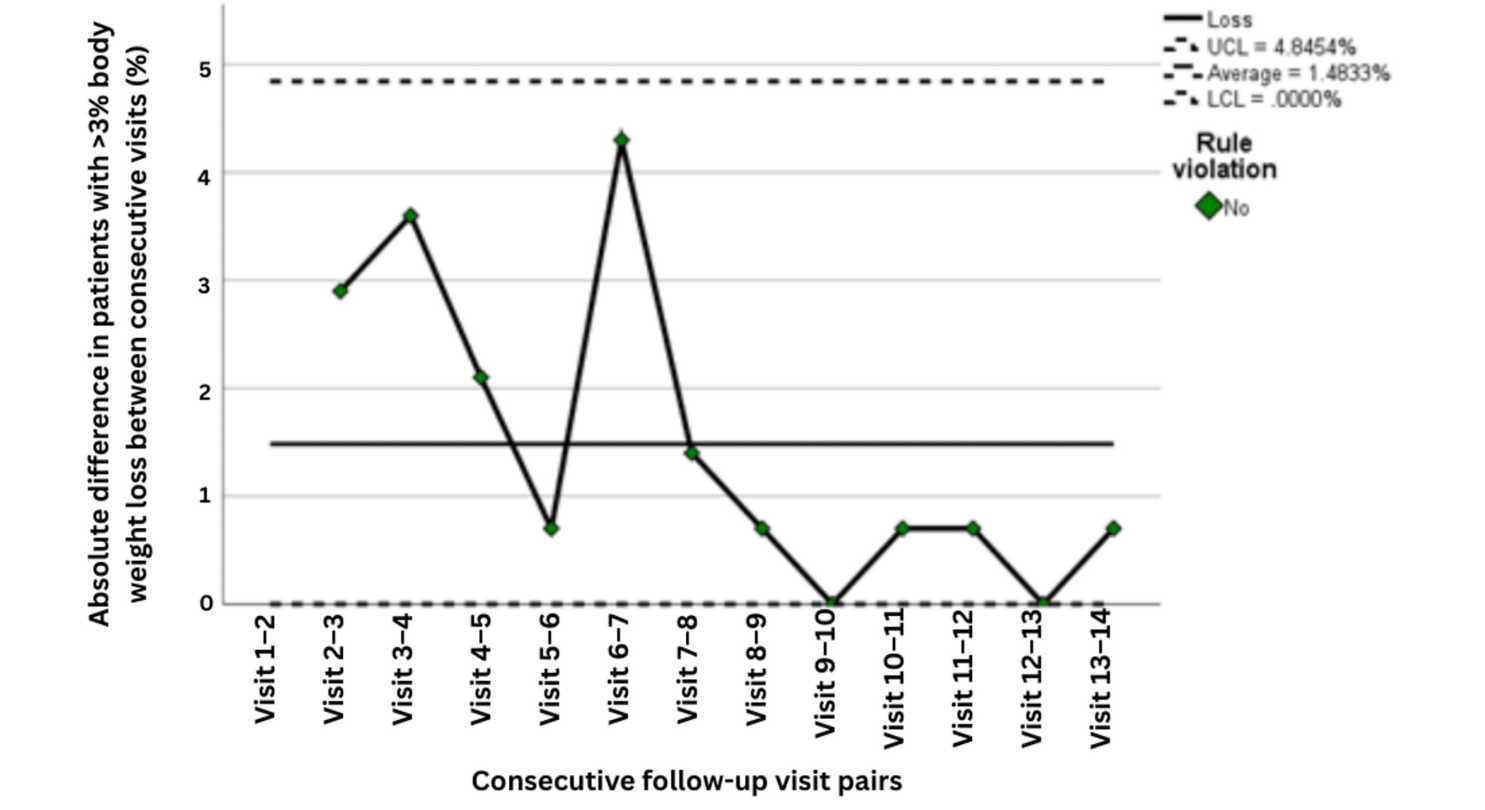
Moving range control chart for visit-to-visit variation in the percentage of patients with >3% body weight loss across follow-up visits (n=141). Moving range control chart showing the absolute visit-to-visit variation in the percentage of patients with >3% body weight loss, based on 141 patients at each time point. The moving range was calculated from pairs of consecutive visits (moving range of 2). Visits 1-5 correspond to PDSA Cycle 1, Visits 6-9 correspond to PDSA Cycle 2, and Visits 10-14 correspond to PDSA Cycle 3. No control chart rule violations were detected, indicating no evidence of special-cause variation. The x-axis represents sequential pairs of consecutive follow-up visits. UCL indicates the upper control limit, and LCL indicates the lower control limit. PDSA: Plan, Do, Study, Act

A parallel analysis was performed for the percentage of patients experiencing >3% weight loss during active treatment. The process capability analysis for this undesirable outcome indicated an overall capable process, with CPa = 2.536, CpLa = 0.899, CpUa = 4.172, and CpKa = 0.899, suggesting that although the process remained within specification limits overall, there was still a relative tendency toward the lower specification boundary, consistent with residual risk of clinically significant weight loss in a subset of patients. These findings support the need for continued nutritional surveillance even after process stabilization (Table 6 and Figures 9-10).

**Table 6:** Process capability indices for clinically significant weight loss (>3%) during follow-up. ^a^ The normal distribution was assumed, with a lower specification limit (LSL) of 0 and an upper specification limit (USL) of 20. The estimated capability sigma was based on the mean of the sample moving ranges. Cp: process capability index; Cpl: lower process capability index; Cpu: upper process capability index; Cpk: minimum process capability index adjusted for process centering

Process statistics		
Capability indices	Cp^a^	2.536
	Cpl^a^	0.899
	Cpu^a^	4.172
	Cpk^a^	0.899

**Figure 11:**
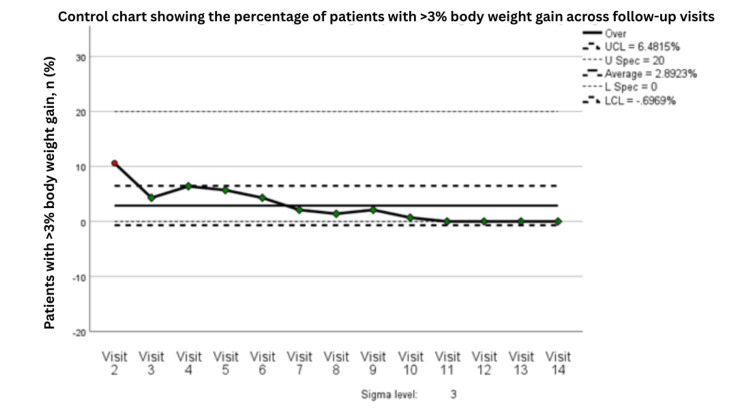
Control chart showing the percentage of patients with >3% body weight gain across follow-up visits (n=141). Statistical process control chart showing the percentage of patients with >3% body weight gain across follow-up visits, based on 141 patients at each time point. Visits 1-3 correspond to PDSA Cycle 1, Visits 4-6 correspond to PDSA Cycle 2, and Visits 7-9 correspond to PDSA Cycle 3. UCL indicates the upper control limit, LCL the lower control limit, U Spec the upper specification limit, and L Spec the lower specification limit. Green points indicate observations within expected process behavior, whereas red points indicate observations flagged by control chart rules as potential special-cause variation. PDSA: Plan, Do, Study, Act

**Figure 12:**
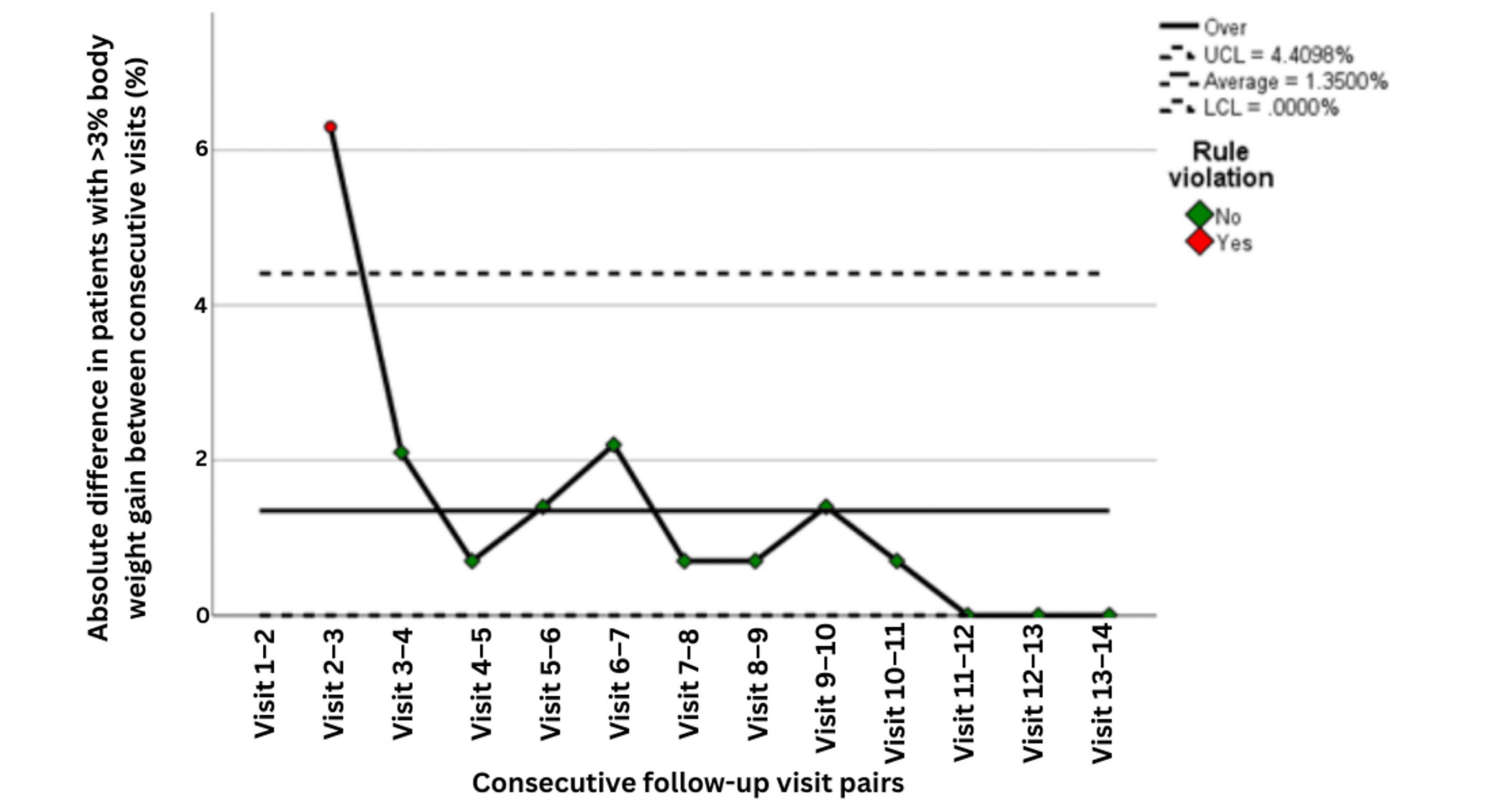
Moving range control chart for visit-to-visit variation in the percentage of patients with >3% body weight gain across follow-up visits (n=141). Moving range control chart showing the absolute visit-to-visit variation in the percentage of patients with >3% body weight gain, based on 141 patients at each time point. The moving range was calculated from pairs of consecutive visits (moving range of 2). Visits 1-5 correspond to PDSA Cycle 1, Visits 6-9 correspond to PDSA Cycle 2, and Visits 10-14 correspond to PDSA Cycle 3. A control chart rule violation was identified early in the series, indicating special-cause variation, followed by a sustained reduction in variability over subsequent visits. The x-axis represents sequential pairs of consecutive follow-up visits. UCL indicates the upper control limit, and LCL indicates the lower control limit. PDSA: Plan, Do, Study, Act

The balancing measure, defined as the percentage of patients experiencing >3% weight gain, also demonstrated acceptable control. The control chart showed that excess weight gain was initially somewhat unstable, with one early point exceeding the upper control limit, but subsequently declined and stabilized within acceptable limits over time (Figure 11). The moving-range chart similarly demonstrated initially high variability followed by sustained reduction and a stable in-control pattern, indicating improved consistency in balancing nutritional support against the risk of excessive gain (Figure 12).

Process capability analysis for the balancing measure showed an overall capable and stable process, with CPa = 2.786, CpLa = 0.806, CpUa = 4.766, and CpKa = 0.806 (Table 7). These findings indicate that while occasional deviations toward undesired weight gain occurred, the intervention pathway maintained good overall balance and did not produce systematic overcorrection. Taken together, the results suggest that the nutritional improvement initiative successfully reduced undesirable weight variation in both directions while preserving process stability and sustained control.

**Table 7:** Process capability indices for clinically significant weight gain (>3%) during follow-up. ^a^ The normal distribution was assumed, with a lower specification limit (LSL) of 0 and an upper specification limit (USL) of 20. The estimated capability sigma was based on the mean of the sample moving ranges. Cp: process capability index; Cpl: lower process capability index; Cpu: upper process capability index; Cpk: minimum process capability index adjusted for process centering

Process statistics		
Capability indices	Cp^a^	2.786
	Cpl^a^	0.806
	Cpu^a^	4.766
	Cpk^a^	0.806

Overall, the findings demonstrate that implementation of a structured, stepwise nutritional support pathway was associated with improved and sustained weight stability among patients receiving active oncologic treatment. The combination of standardized education, selective escalation to ONS, and pharmacologic support for refractory cases was accompanied by progressive reduction in process variation, strong capability performance, and maintenance of balance without excessive weight gain.

Patient-reported satisfaction was high across the intervention domains. For nutritional education, overall satisfaction was high, with 122 (86.5%) patients reporting being very satisfied and 19 (13.5%) satisfied. For ONS, overall satisfaction was 104 (73.8%) very satisfied and 28 (19.9%) satisfied, while nine (6.4%) patients expressed dissatisfaction related to taste and tolerance. For the overall clinical nutrition service, 122 (86.5%) patients were very satisfied, and 19 (13.5%) were satisfied, with no dissatisfied responses reported. These findings indicate that the intervention was highly acceptable to patients and was perceived as clear, practical, and supportive during active treatment.

## Discussion

Summary

In this quality improvement project involving 141 patients receiving active oncologic therapy, implementation of a structured integrative nutritional support pathway was associated with a marked and sustained improvement in weight stability during treatment. All patients received nutritional education, establishing a uniform foundation for care delivery. However, 58.2% required ONS, indicating that education alone was frequently insufficient in the context of treatment-related toxicities, appetite suppression, and altered metabolism. Overall, 75.2% of patients were compliant with the nutritional plan and maintained stable body weight, whereas a proportion of non-compliant or persistently symptomatic patients required further escalation to pharmacologic support, including olanzapine, to address anorexia and treatment-related nausea and vomiting. These findings are consistent with the project rationale and specific aim of reducing clinically meaningful weight loss through structured and escalating nutritional support (Table 5 and Figure 8).

The process-control findings further strengthen the relevance of these results. Weight-stability rates improved from approximately 80% during earlier visits to above the process average of 93.6% by Visit 7, with sustained performance thereafter near the upper control limit. The moving-range chart demonstrated declining variability over time, suggesting that the intervention not only improved outcomes but also reduced process instability. Capability indices (Cp = 2.809, Cpk = 2.541) were well above the conventional benchmark of 1.33, indicating a highly capable and reliable process. Together, these results suggest that the intervention achieved meaningful and durable improvement in routine clinical care rather than a transient or unstable effect (Figures 9-10 and Table 6).

A particular strength of this project was the use of a structured, multidisciplinary, and stepwise escalation model. The pathway moved logically from universal education to targeted ONS to pharmacologic support in selected refractory cases, while being continuously monitored using statistical process control (SPC). This design allowed the intervention to remain pragmatic, responsive, and embedded within existing oncology workflows, which likely contributed to both uptake and sustainability.

Interpretation

The observed association between the intervention bundle and improved weight outcomes is clinically plausible and aligns with the intended mechanism of the project. The pathway addressed several major determinants of cancer-related nutritional decline simultaneously: inadequate intake, delayed nutritional escalation, treatment-related symptoms, and barriers to adherence. Early screening and standardized counselling created a baseline level of support for all patients, while escalation to ONS and pharmacologic management allowed higher-risk individuals to receive more intensive and individualized intervention. In this sense, the project functioned not merely as a nutritional add-on but as a systems-based supportive care strategy that stabilized risk early and reduced avoidable deterioration over time.

These findings are consistent with previous literature showing that ONS can reduce weight loss in cancer patients. Meta-analytic evidence has shown that patients receiving ONS lose less weight than controls [16]. Likewise, Qin et al. demonstrated that ONS combined with nutrition education improved nutritional risk scores and biochemical nutritional indicators compared with education alone in patients undergoing chemotherapy [17]. Nett et al. also reported that structured nutritional intervention pathways incorporating counselling, supplementation, and dietitian support reduced postoperative weight loss and improved nutritional outcomes in oral cancer patients [18]. A more recent meta-analysis and systematic review similarly found that structured nutrition interventions improve quality of life, reduce fatigue, enhance protein and energy intake, and reduce unplanned hospitalizations during chemotherapy or radiotherapy [19,20].

Our adherence findings are also broadly consistent with prior work. Adherence to ONS is often suboptimal in real-world oncology care [21,22]. In the present project, the 75.2% compliance rate can be considered relatively favourable and may reflect the close follow-up, repeated reinforcement, and multidisciplinary oversight built into the quality improvement framework. This interpretation is supported by reports from Liljeberg et al., who demonstrated higher adherence when patients were able to taste supplements and choose preferred flavours [23]. It is therefore plausible that adherence in our cohort could have been further improved through systematic incorporation of flavour choice and palatability testing. Similarly, evidence suggesting that high-protein, low-volume formulations may better support protein intake in oncology patients [24] may help explain why a more tailored supplement strategy could enhance future results even further.

The impact of this project extends beyond numeric improvement in weight stability. For patients, nutritional stabilization may help preserve functional reserve, reduce treatment interruptions, improve treatment tolerance, and enhance quality of life. From the health-system perspective, a more stable and predictable nutritional process may reduce reactive escalation, decrease care variability, and support more efficient allocation of supportive care resources. Because poor nutritional status in oncology is associated with higher complication rates, more unplanned admissions, and shorter survival [25], embedding nutritional surveillance into routine oncology practice may have broad downstream benefits for both patient outcomes and service performance.

In addition to improving weight-related outcomes, the intervention was highly acceptable to patients, with consistently high satisfaction across nutritional education, ONS, and the overall clinical nutrition service. These findings support the patient-centeredness and practical acceptability of the pathway, although satisfaction should be interpreted as a complementary finding rather than a substitute for clinical effectiveness.

One notable feature of this project was the integration of SPC into supportive oncology care. This enabled real-time monitoring of both improvement and stability and provided a practical framework for identifying whether gains were sustained rather than episodic. The process appeared to outperform expectations, achieving faster-than-anticipated convergence into control, higher-than-expected capability indices, and minimal regression after stabilization. Nonetheless, some patients remained vulnerable, with weight trajectories approaching the lower specification limit. This likely reflects the biological limits imposed by advanced disease, tumor burden, inflammation, sarcopenia, and aggressive treatment regimens. Accordingly, while the aggregate outcomes were strongly favourable, individual-level variation underscores the need for continued personalization and escalation for high-risk subgroups.

The project also involved practical trade-offs. Early dietitian involvement, frequent reassessment, repeated counselling, and ongoing data monitoring likely increased workload and required sustained interdisciplinary engagement. Although these inputs appear justified by the observed gains, they raise important considerations regarding staffing, operational capacity, and long-term integration into standard workflows. In this sense, the project demonstrates not only clinical feasibility but also the need to align nutritional improvement work with institutional support and quality infrastructure.

From a practical perspective, the findings support the implementation of a simple stepwise nutritional pathway in oncology settings. This pathway should begin with routine weight measurement and nutritional risk review at each treatment visit, followed by standardized nutrition education for all patients. Patients with ≥3% weight loss, reduced intake, poor appetite, or significant nutrition-impact symptoms should receive early dietitian review and individualized ONS. Patients who continue to lose weight or report persistent nausea, vomiting, anorexia, or psychological barriers should be escalated for physician-led symptom management and supportive care. In settings with limited resources, the same model can be adapted using basic weight tracking, structured counselling checklists, prioritized dietitian referral for high-risk patients, and periodic review of weight-stability rates as a quality indicator.

Limitations

Several limitations should be acknowledged. First, patient adherence was not universal; some patients refused to follow nutritional instructions, which likely limited the full benefit of the intervention. Second, elderly patients often attended treatment without family members or caregivers who could reinforce recommendations or support adherence at home. Third, early in the project, some departments lacked weighing scales, which may have delayed baseline or follow-up measurements and introduced variability in data capture. Fourth, psychological support was not systematically integrated, meaning that patients with depression, anxiety, or distress may have been less able to engage fully with nutritional recommendations.

Additional limitations relate to implementation and interpretation. Early dietitian involvement proved feasible and effective, but it also increased workload, raising questions about long-term staffing sustainability. Some patients declined ONS because of taste intolerance, suggesting that greater variety and more flexible formulation strategies may be needed. Moreover, not all weight loss in oncology is preventable, particularly in patients with advanced cachexia or aggressive disease biology. Outcomes should therefore be interpreted within this biological context rather than assumed to be fully modifiable. Finally, data quality depended on accurate clinical documentation, and occasional omissions may have introduced measurement bias or limited internal validity.

An additional limitation is the absence of a concurrent comparator or control group. Because this was a single-center quality improvement project embedded in routine care, the before-and-after design limits causal inference. Although the temporal association, sequential PDSA implementation, and statistical process-control findings support an association between the intervention pathway and improved weight stability, improvements cannot be attributed solely to the intervention. Secular trends, increased clinical attention, changes in patient engagement, or other unmeasured service factors may also have contributed to the observed outcomes.

Despite these limitations, several aspects of the project helped mitigate their impact. The pathway was standardized, multidisciplinary, and iteratively refined through PDSA cycles; data were monitored longitudinally rather than at a single time point; and SPC methods allowed assessment of both outcome improvement and process stability. These features strengthen confidence that the observed gains were meaningful at the service level, even if some individual-level confounding and implementation variation remained.

## Conclusions

This project demonstrates that implementation of a structured, multidisciplinary nutritional support program can significantly improve and sustain weight stability among cancer patients receiving active treatment. The pathway achieved statistical control, high process capability, and favourable adherence, supporting its role as a practical and effective quality improvement strategy in oncology care. By integrating nutritional screening, standardized counselling, ONS, escalation pathways, and real-time monitoring, the intervention reframed nutrition as a core component of patient safety, treatment continuity, and person-centered cancer care rather than as supportive care alone. The high level of patient satisfaction further supports the acceptability and feasibility of this model within routine oncology practice. The work also appears highly sustainable and potentially transferable. In line with the NHS Sustainability Model described in the source document, the intervention benefited from visible leadership support, multidisciplinary engagement, process adaptability, and alignment with routine care structures. Standardized screening tools, educational materials, and process-control dashboards are all elements that can be embedded into existing electronic or quality systems with relatively modest additional resource requirements. As such, this model may be adaptable not only across oncology centers and radiotherapy units but also to other settings in which malnutrition materially affects outcomes, including surgical, chronic disease, and critical care services.

From a practice perspective, these findings support the incorporation of nutritional screening and escalation into every treatment cycle as a measurable quality indicator in oncology. From a research perspective, future work should examine cost-effectiveness more directly, including hospitalization, toxicity, treatment interruptions, and survival outcomes according to nutritional stability. Further evaluation of psycho-oncological integration, taste-adapted supplement strategies, and digital adherence platforms may also help optimize patient engagement and strengthen the evidence base for widespread implementation.
